# Tar Barreler's Hump: An Unusual Presentation of a Posttraumatic Pseudolipoma

**DOI:** 10.1155/2012/130973

**Published:** 2012-08-16

**Authors:** Babajide Olusola Olubaniyi, Harbir Sidhu, Alex Long, Nigel de-Sousa, Andrew Redfern

**Affiliations:** ^1^Peninsula Radiology Academy, Plymouth International Business Park, Plymouth PL6 5WR, UK; ^2^Department of Radiology, Royal Devon and Exeter Hospital, Barrack Road, Exeter EX2 5DW, UK; ^3^Coleridge Medical Centre, Canaan Way, Ottery St. Mary, Devon EX11 1EQ, UK

## Abstract

This is an interesting paper of a 4 cm posttraumatic pseudolipoma on the back of the neck of an adult man who has participated in “tar barrel rolling” since adolescence. To the best of our knowledge, this is the first case of a pseudolipoma to be reported in the literature in association with tar barreling.

## 1. Case Presentation

**Figure 1:**
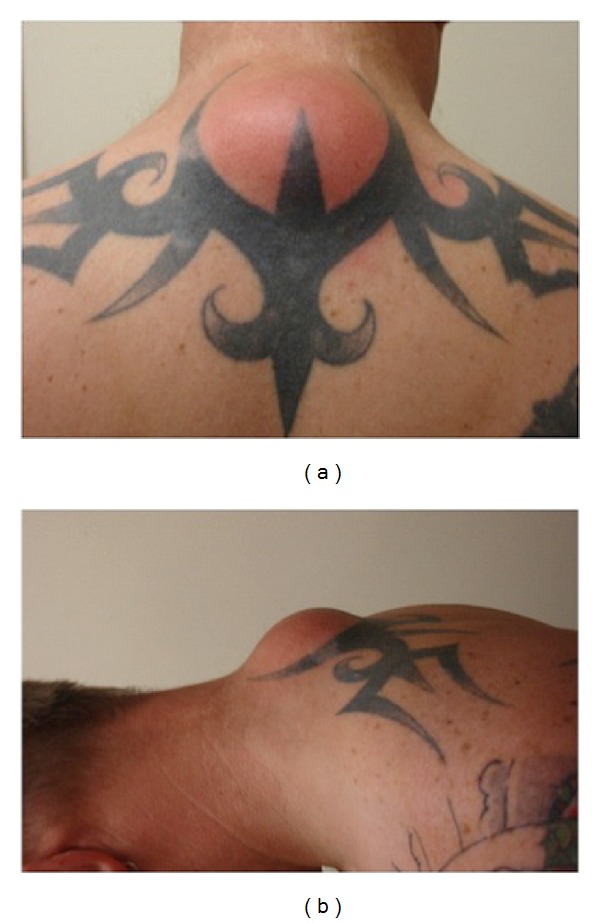
4 cm nontender midline soft tissue mass in the interscapular region in a 37-year-old male “tar barreler.”

A 37-year-old healthy man known to have a “tar barreler's hump” in his local community presented to his general practitioner because his hump had become acutely inflamed. The hump has been present for over fifteen years without causing any symptoms. He has participated in “tar barrel rolling”—a family tradition since adolescence. Examination of his back revealed a 4 cm nontender solid soft tissue mass at the posterior aspect of the lower neck in the interscapular region, there was mild erythema of the overlying skin (Figure 1). No other lumps were found.

**Figure 2:**
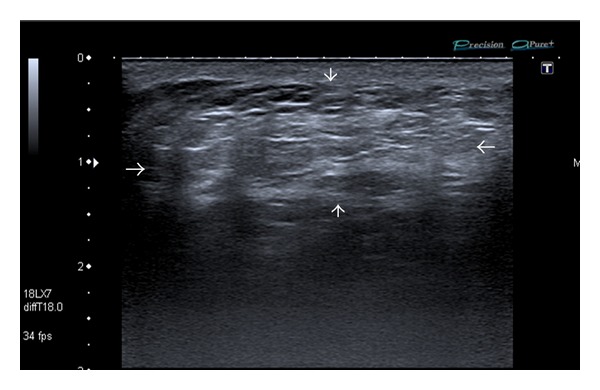
Ultrasound examination (longitudinal view) demonstrates a large subcutaneous mass (bounded by white arrows) in the interscapular region with sonographic features consistent with a lipoid mass, that is, elliptical shape, heterogeneity, longest axis parallel to the skin surface, lack of posterior acoustic enhancement or attenuation, and presence of multiple echogenic lines perpendicular to the ultrasound beam.

**Figure 3:**
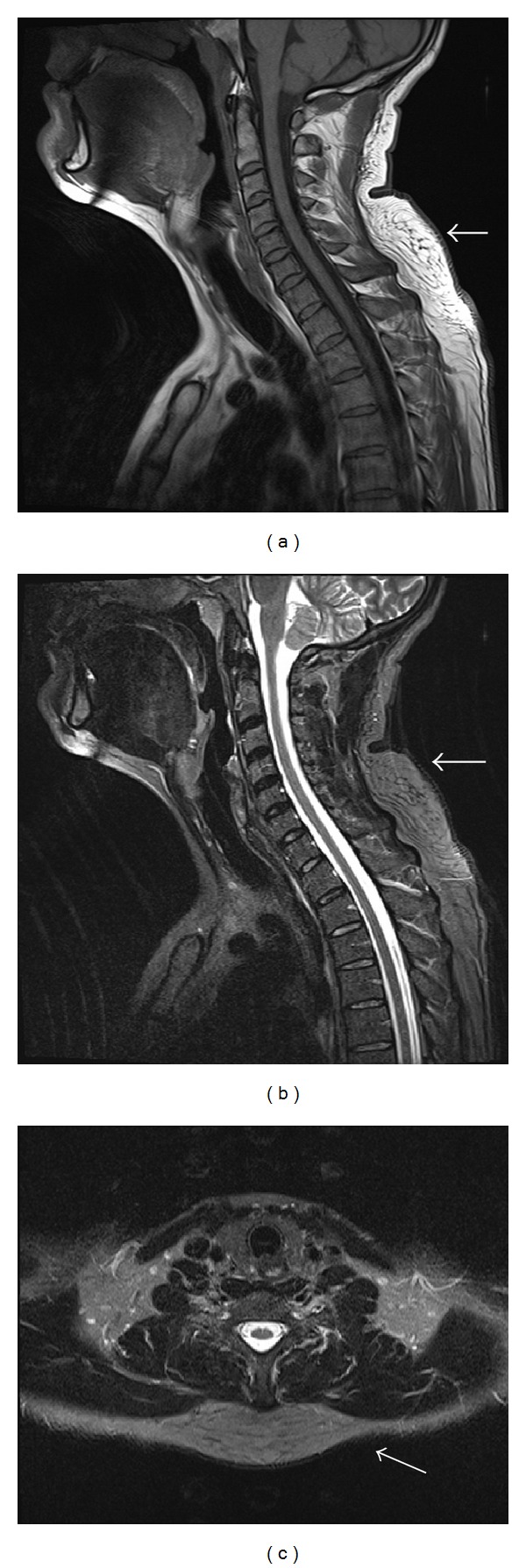
MRI of the neck demonstrates a subcutaneous mass (white arrows) in the interscapular region isointense to surrounding subcutaneous fat on T1-weighted sequence (Figure 3(a), repetition time/echo time = 451/11 msec) and on fat-suppressed sequence (Figures 3(b) and 3(c), repetition time/echo time = 7090/89 msec). There is absence of a low signal intensity fibrous capsule (Figure 3(c)) typical of a lipoma.

Ultrasound examination (Toshiba Aplio XG, 7.5 MHz probe) revealed a hyperechoic mass in the subcutaneous tissues of the interscapular region (Figure 2). Due to the history of recent onset of pain, magnetic resonance imaging (MRI) was performed for further assessment. MRI confirmed an unencapsulated subcutaneous mass that is isointense to surrounding fat on both T1-weighted (T1W1) and fat-suppressed sequences (Figure 3) consistent with a benign posttraumatic pseudolipoma (PTL). The patient declined referral to the plastic surgeon for further management. Subsequent followup by the referring general practitioner revealed complete resolution of the associated mild inflammation around the pseudolipoma following completion of a course of simple analgesia. 

## 2. Discussion

“Tar barreling” is a world famous tradition native to Ottery St Mary, Devon, Southwest England. The tradition dates back to the 17th century, and it is performed annually on the 5th of November (Guy Fawkes Night). Barrels soaked in tar are set ablaze and carried on the back between the shoulders through the streets. Different categories exist for boys, women, and men depending on the size of the barrels. The event culminates at night with men carrying flaming barrels that weigh up to 30 kg. The festival attracts between 15–20,000 visitors annually. 

In a few cases, generations of the same family carry these flaming tar barrels annually; men often start “barrel rolling” at a very young age similar to the case presented. Allegedly, there are a few participating residents in the community who have also developed humps at the back of the neck where these barrels are carried over the years. These humps are known in the local community as “tar barreler's hump” and are regarded to be of no serious medical significance. 

Although there is lack of histological analysis in our case as surgical management was declined, the appearance on ultrasound that is, well-delineated hyperechoic subcutaneous mass with linear echogenic lines perpendicular to the ultrasound, beam and absence of posterior acoustic enhancement or attenuation are typical sonographic appearance of a lipoma or lipoma-like lesion [2]. MRI appearances that is, isointensity to fat on T1-weighted and fat-suppressed sequences are also consistent with a lipoid lesion [3]. However, the absence of a well-defined low signal intensity fibrous capsule on MRI along with the history of repetitive trauma is diagnostic of a posttraumatic pseudolipoma (PTL). 

PTLs are benign soft tissue tumours that develop in various anatomical regions following acute, chronic or repetitive trauma. The exact pathobiological mechanism of development of PTL is unclear; however various theories via mechanical and inflammatory factors have been postulated. Mechanical factors such as herniation of fatty tissue following traumatic disruption of fascial layers or differentiation of preadipocytes triggered by growth factors and inflammatory mediators such as cytokines released from the preceding haematoma are some of the postulated mechanisms [1, 4].  Table 1 summarises the clinical and imaging features of PTLs. Although benign, PTLs can enlarge rapidly or cause local symptoms that may warrant cross-sectional imaging as in our case to ensure benignity.

Imaging can be used to differentiate benign lipomatous tumours from liposarcomas [5–8]. The typical appearance of PTL, lipoma, and liposarcoma is summarised in Table 2. PTLs are homogenous masses with signal intensity identical to surrounding fat on all pulse sequences; they lack a low signal intensity fibrous capsule typical of lipoma, and there is no postcontrast enhancement [1, 4]. Definitive management depends on their location, size, or associated symptoms and treatment options include surgical excision or liposuction.

We cannot speculate the natural progression of this lesion however malignant transformation into liposarcoma has never been reported as sequelae of PTLs. To the best of our knowledge, this is the first case of PTL in association with tar barrelling to be reported in published the literature.

## Figures and Tables

**Table 1:** Summary table: posttraumatic pseudolipoma (PTL).

Etiology	Sequelae of acute, chronic, or repetitive trauma. Various mechanisms of development are postulated
Incidence	~1%
Gender ratio	F : M = 3.8: 1^∗^
Age	18–64 years^∗^
Risk factors	Acute/chronic/repetitive trauma
	Conservative
Treatment	Liposuction
	Surgical excision
Prognosis	Unknown. Malignant transformation has never been reported
Imaging	US—well-delineated hyperechoic subcutaneous mass, no posterior acoustic attenuation or enhancement
	CT—subcutaneous mass with Hounsfield attenuation of fat
	MRI—homogenous unencapsulated mass isointense to fat on all sequences
	T1WI-hyperintense (similar to fat)
	Fat-suppressed sequence—hypointense (similar to fat)
	Lack of a well-defined low signal intensity fibrous capsule
	Lack of enhancement following administration of intravenous contrast

^∗^Based on a review of 124 cases of PTLs by Galea et al. [1].

**Table 2:** Typical imaging features of PTL, lipoma, and liposarcoma.

	US	CT	MRI
Posttraumatic pseudolipoma (PTL)	Well-delineated hyperechoic subcutaneous mass	Well-delineated subcutaneous mass with Hounsfield attenuation of fat	Homogenous unencapsulated mass isointense to fat on all sequences
	No posterior acoustic attenuation or enhancement		T1WI-hyperintense
			T2WI-hypointense
			Fat suppression—hypointense
			Absence of a well-defined low signal intensity fibrous capsule
			No enhancement following administration of intravenous Gadolinium

Lipoma	Similar to PTL	Similar to PTL	Signal intensity as PTL
		Usually homogenous	Usually homogenous
		May contain thin internal septa (<2 mm)	A well-defined low signal intensity fibrous capsule is usually present
		May appear as complex with thick septa (>2 mm) and nonlipomatous components	There may be mild to moderate enhancement following administration of intravenous gadolinium

Liposarcoma (variable appearance according to histological type according to WHO classification	Heterogeneous, multilobulated usually well-defined fatty mass	Contains large lipomatous and prominent nonlipomatous components such as thick internal septa (>2 mm)	Heterogeneous Contains large lipomatous and prominent nonlipomatous components such as thick internal septa (>2 mm) and nodules
	Usually located in the deep soft tissues of the extremities particularly thigh, head and neck, trunk, and retroperitoneum	May contain focal nodules	Moderate to marked enhancement of septa following administration of intravenous gadolinium
		Calcification or metaplastic ossification may be seen	
